# 

**Published:** 1879-02

**Authors:** 


### CONTENTS.

Why do Fillings Fail. *By Wm. A. Pease, Dayton, O*.......... .......... 53
Can Dental Lesions be Foreseen and Prevented. *By De-Ce-Aich*...... 76
Have we Not too Many Dental Schools? *Dow 3d*........................ 76
PROCEEDINGS OF SOCIETIES.
American Academy of Dental Science .......................................... 78
Proceedings of the Tennessee Dental Society................................ 82
MISCELLANEOUS.
Prof. Ashurst on Anæsthetics .................................................. 91
An Anomalous Case of Replantation. *By A. W. Harlan, Chicago, Ill.* 92
EDITORIAL.
Bibliographical.................................................................. 93
Mississippi Valley Dental Association........................................ 94
California State Dental Association........................................... 5
Meeting............................................................................ 95
Commencement.................................................................. 96
Alumni.............................................................................. 96

